# Supplementary material for: Determination of radiocarbon in environmental objects
Source: PLoS One. 2025 Jun 5;20(6):e0324818. doi: 10.1371/journal.pone.0324818 (PMC12140196; doi:10.1371/journal.pone.0324818)
Supplement: S1 Fig — (DOCX) [file pone.0324818.s001.docx]

| **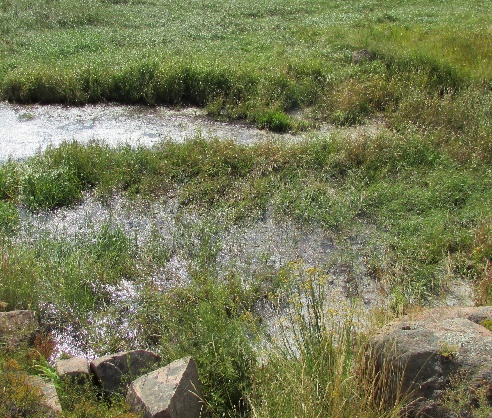**  **tunnel 104** | 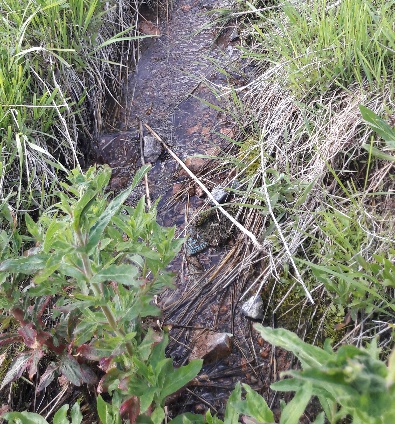  **tunnel 165** | | 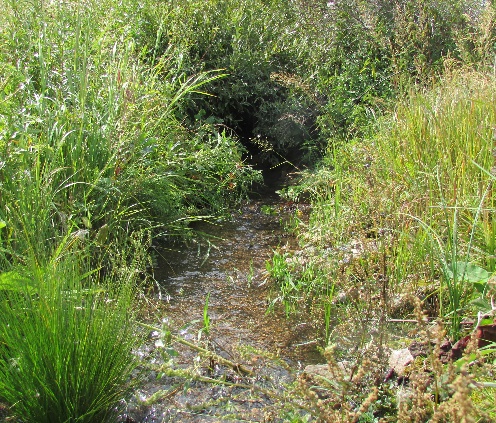  **tunnel 177** |
| --- | --- | --- | --- |
| 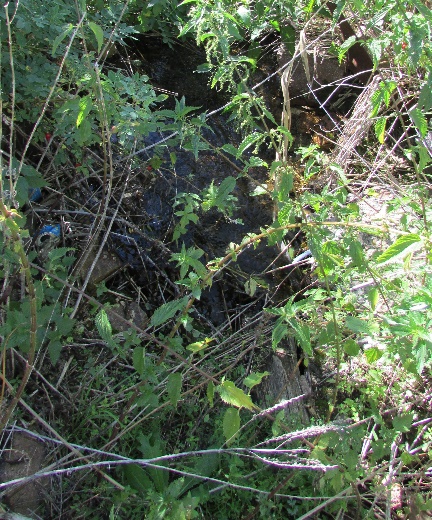  **tunnel 176** | | 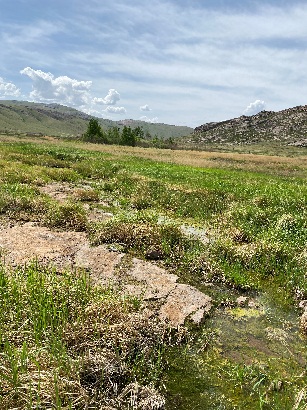  **tunnel 504** | |
| **Fig 1. Research areas at the “Degelen” test site** | | | |

| **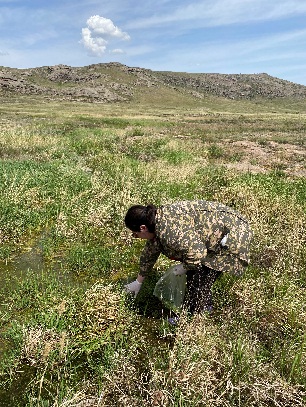** | **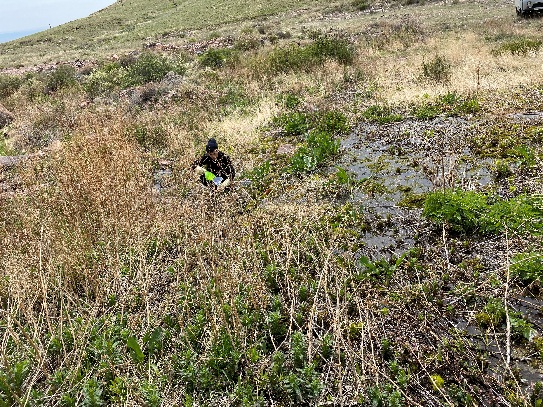** | **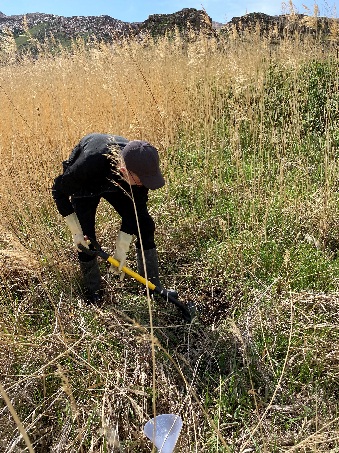** |
| --- | --- | --- |
| **Fig 2. Sampling of plants, water and soil** | | |

| 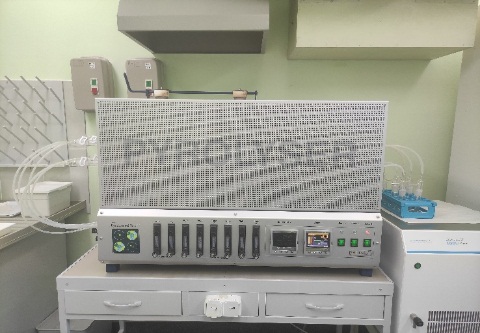 | 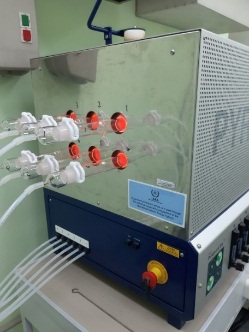 | 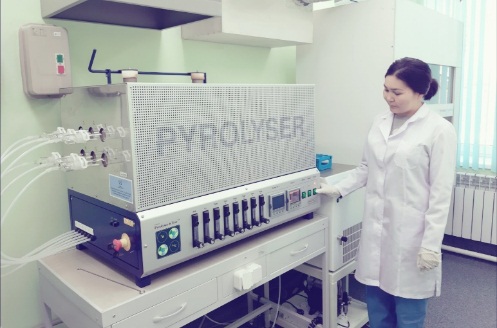 |
| --- | --- | --- |
| **Fig 3. Pyrolyser-6 Trio** | | |

| 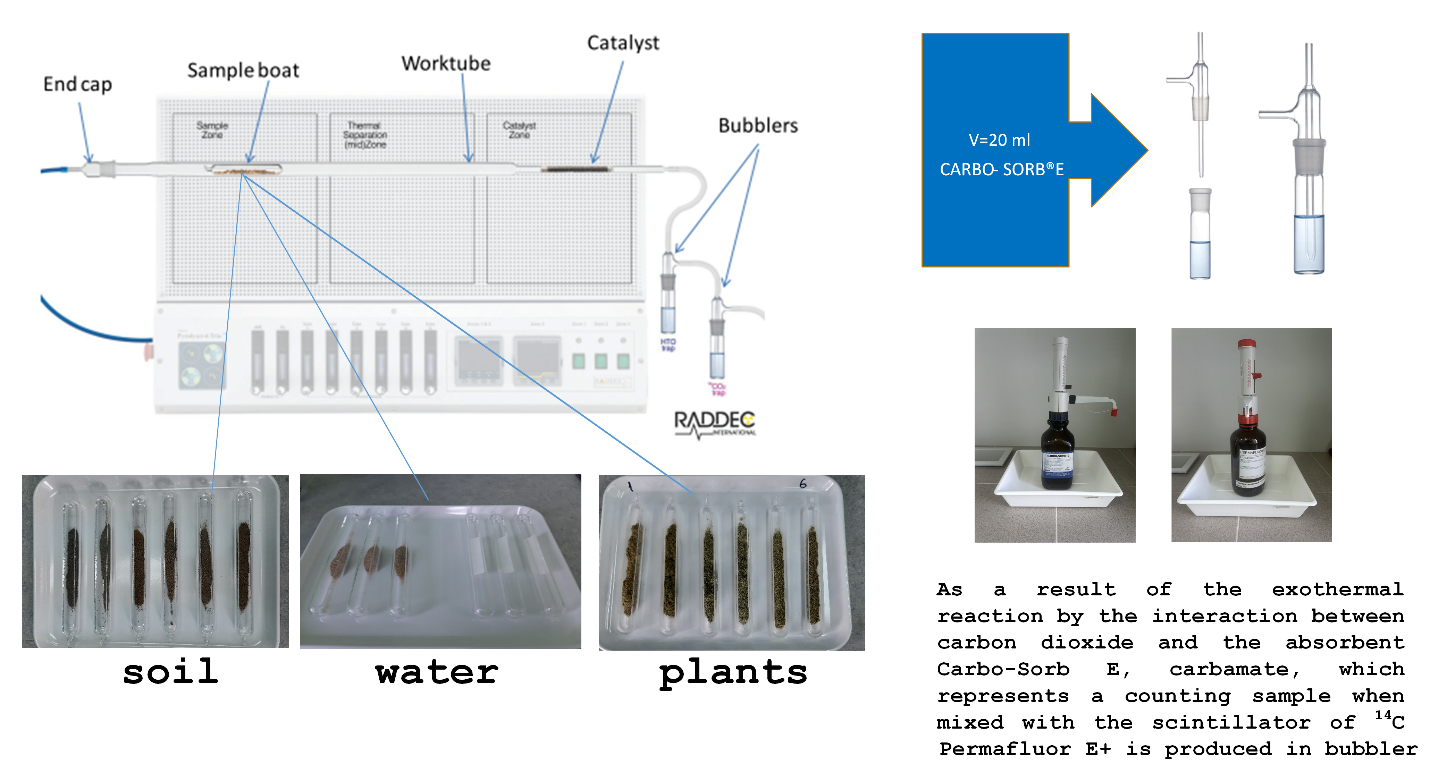 | | |
| --- | --- | --- |
| **Fig 4. Counting sample preparation** | | |
|  | | |
| 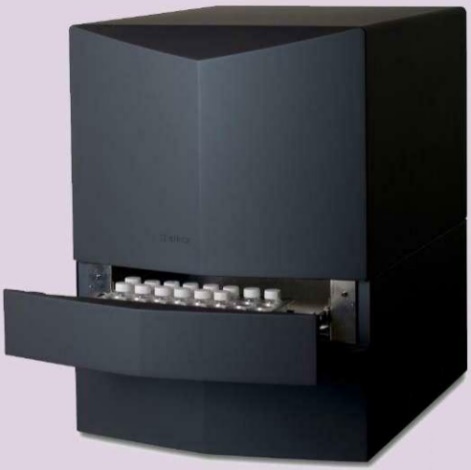 | 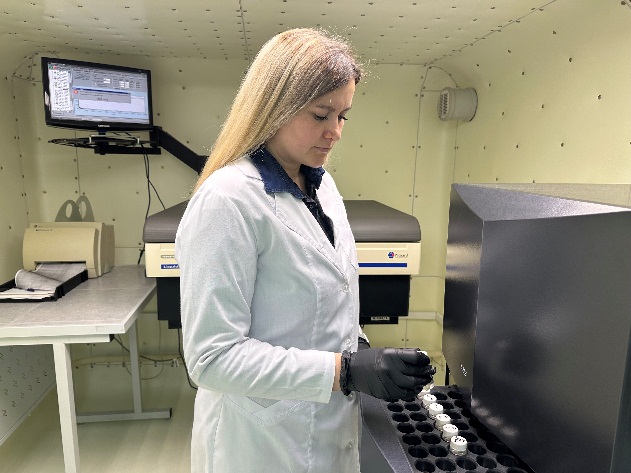 |  |
| **Fig 5. Highly sensitive alpha-beta radiometer SL-300** | |  |

| 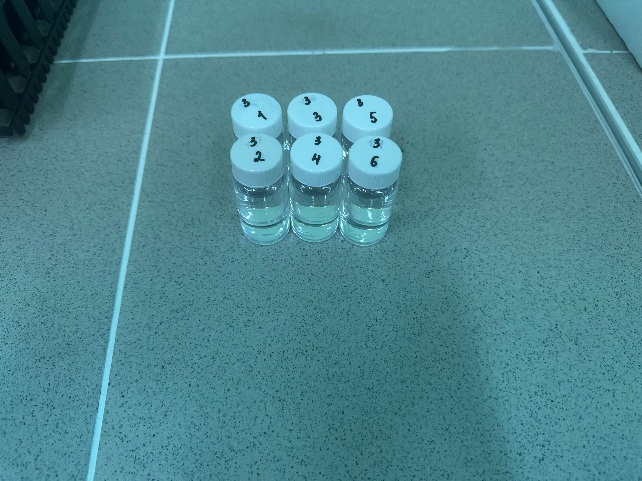 |
| --- |
| **Fig 6. Counting samples** |
