# Supplementary material for: Determination of radiocarbon in environmental objects
Source: PLoS One. 2025 Jun 5;20(6):e0324818. doi: 10.1371/journal.pone.0324818 (PMC12140196; doi:10.1371/journal.pone.0324818)
Supplement: S1 File — (PDF) [file pone.0324818.s002.pdf]

Request for Permission to Publish Content under CC-BY License

Dear Rights Holder or Representative,

I have submitted a paper for publication in a PLOS journal, and wish to include the content listed below in the paper. I'm hereby requesting your (or your company's or institution's) permission to include the content in my paper. Please note that all PLOS journals are published under a Creative Commons Attribution License (CC BY), which allows for unrestricted use and distribution, even commercial, as long as attribution is given to the creator or rights holder of the content. See https://creativecommons.org/licenses/by/4.0/.

To grant me permission to use the content in my PLOS paper, please fill in the information below and then scan the completed form and send it to me at my email address.

Thank you.

My name:

Mariya T. Abisheva

My email address:

Abisheva@nnc.kz

Description of the content which I'm seeking permission to use (citation and/or title, and pasted screen shot, if applicable):

Fig 1. Sampling points of environment objects

Link to the Content:

https://creativecommons.org/licenses/by/4.0/?ref=chooser-v1

* * *

On behalf of myself or the rights holder, I hereby grant the permission sought herein.

Signature of Party Granting Permission:

Date:

February 26, 2025

Printed Name and Title:

Mariya T. Abisheva, Head of the Information Support Department
Branch office 'Institute of Radiation Safety and Ecology'
Republican State Enterprise 'National Nuclear Center of the Republic of Kazakhstan'
