# Supplementary material for: Determination of radiocarbon in environmental objects
Source: PLoS One. 2025 Jun 5;20(6):e0324818. doi: 10.1371/journal.pone.0324818 (PMC12140196; doi:10.1371/journal.pone.0324818)
Supplement: S2 File — (PDF) [file pone.0324818.s003.pdf]

Search Donate Explore CC

creative commons

WHO WE ARE WHAT WE DO LICENSES AND TOOLS BLOG SUPPORT US

Home > Chooser

LICENSE CHOOSER

Follow the steps to select the appropriate license for your work. This site does not store any information.

1 **Do you know which license you need?**
I know the license I need.

2 **Attribution**
Creative Commons Attribution 4.0 International

3 **Commercial Use**

Filling out this form is optional, but helps others attribute your work to you, and fills in machine-readable code.

Title of Work

Fig 1. Sampling points of environment objects

Creator of Work

Mariya T. Abisheva

Link to Work

https://janedoe.com/best-photo-ever.jpg

Link to Creator Profile

Abisheva@nnc.kz

Year Of Creation

2025

BACK DONE Start again

RECOMMENDED LICENSE

CC BY 4.0

Creative Commons Attribution 4.0 International

This license requires that reusers give credit to the creator. It allows reusers to distribute, remix, adapt, and build upon the material in any medium or format, even for commercial purposes.

BY: Credit must be given to you, the creator.

See the License Deed

MARK YOUR WORK

Choose the kind of work to get appropriate license code or public domain marking.

Website **Print Work or Media**

Copy the text below and paste it on the title and/or copyright page of your print work or presentation, or in the credits of your media.

Plain Text

This work © 2 by M is licensed under CC BY 4.0. To view a copy of this license, visit https://creativecommons.org/licenses/by/4.0/

license abbreviation full license name Copy

CONFUSED? NEED HELP?

- What Are Creative Commons Licenses?
- How to apply a Creative Commons license?
- What should I consider?

- What do the Icons Mean?
- What if I have other questions?

Contact Newsletter Privacy Policies Terms

CONTACT US

Creative Commons
PO Box 1866, Mountain View, CA
94042

info@creativecommons.org

SUBSCRIBE TO OUR NEWSLETTER

Your email SUBSCRIBE

Except where otherwise noted, content on this site is licensed under a Creative Commons Attribution 4.0 International license. Icons by Font Awesome.

SUPPORT OUR WORK

Our work relies on you! Help us keep the Internet free and open.

DONATE NOW
