# Supplementary material for: Determination of radiocarbon in environmental objects
Source: PLoS One. 2025 Jun 5;20(6):e0324818. doi: 10.1371/journal.pone.0324818 (PMC12140196; doi:10.1371/journal.pone.0324818)
Supplement: Attachment — Submitted filename: Response to Reviewers.docx [file pone.0324818.s005.docx]

List of corrections

| **#** | **Comments** | **Explanations** | **New version** |
| --- | --- | --- | --- |
|  | **Reviewer 1** | | |
| 1 | Sentence to be rephrased (Page.2, Line no.39)  “Carbon is a foundamental brick of all life forms on Earth. It is present in the atmosphere, flora, fauna, non-living organic matter, fossil fuel, rock and is dissolved in the ocean.” | Corrected | “Carbon on Earth is found in the atmosphere, flora, fauna, non-living organic matter, fossil fuel, rock and is dissolved in the ocean.” |
| 2 | Kindly provide sample collection season | Added | “Duplicate samples of water, soil and plants were collected in the vicinity of interest water streams in the summer” |
| 3 | What is this HTO (Page.7, Line no.175) | Added | tritiated water (HTO) |
| 4 | In Table 3 Activity Concentration measured Bq/kg for Plants and Soil, but Water Activity Concentration measured Bq/1 . what it indicate | Answer to comment | For solid samples, the Activity Concentration was calculated per 1 mass (per 1 kg of plants, 1 kg of soil). For water, the Activity Concentration was calculated per 1 volume of sample (per 1 liter of water). |
| 5 | In fig.4, What is the significant role shows the same value of 0.7 percentage distribution of ^14^C in tunnel 104 and tunnel 504 | Added | The same value of 0.7 percentage distribution of ^14^C in tunnel 104 and tunnel 504 shows only the similar nature of ^14^C contamination and the same type of soils in the study area. |
| 6 | How Much distance between the tunnel 104 and tunnel 504 | Answer to comment | The distance between the tunnels is about 9 km, and they are located on opposite sides of the Degelen mountain: the tunnel 104 is located on the south-eastern side, the tunnel 504 - on the northern side. |
| 7 | Which of the sampling place is more suitable for ecosystems | Added | L. 328-333 |
| **Reviewer 2** | | | |
| 8 | Clarify ambiguous terms and improve phrasing for better readability (e.g., rephrasing long sentences in the Results section for clarity). | Corrected |  |
| 9 | Include additional statistical analysis, such as significance testing, to validate differences in radiocarbon concentrations across sites and matrices. | Added | L. 246-256 |
| 10 | Expand the discussion in the abstract and conclusion to highlight the novelty of using the Pyrolyser-6 Trio system for C-14 determination and its broader implications for environmental monitoring in radioactively contaminated areas. | Added | L. 32-40, L. 350-355 |
| 11 | Provide access to raw data or deposit it in a public repository to comply with PLOS ONE’s data availability policies. | Added | Additional file |

2. In your Methods section, please provide additional information regarding the permits you obtained for the work. Please ensure you have included the full name of the authority that approved the field site access and, if no permits were required, a brief statement explaining why.

No permit for field work was required, since the “National Nuclear Center of the Republic of Kazakhstan” is the authorized body at the territory of the Semipalatinsk nuclear test site, where the “Degelen” test site is located.
